# Protecting enzymatic function through directed packaging into bacterial outer membrane vesicles

**DOI:** 10.1038/srep24866

**Published:** 2016-04-27

**Authors:** Nathan J. Alves, Kendrick B. Turner, Igor L. Medintz, Scott A. Walper

**Affiliations:** 1 National Research Council, 500 Fifth Street NW (Keck 576), Washington, DC 20001, USA; 2 Center for Bio/Molecular Science & Engineering, US Naval Research Laboratory, Washington, DC 20375, USA

## Abstract

Bacteria possess innate machinery to transport extracellular cargo between cells as well as package virulence factors to infect host cells by secreting outer membrane vesicles (OMVs) that contain small molecules, proteins, and genetic material. These robust proteoliposomes have evolved naturally to be resistant to degradation and provide a supportive environment to extend the activity of encapsulated cargo. In this study, we sought to exploit bacterial OMV formation to package and maintain the activity of an enzyme, phosphotriesterase (PTE), under challenging storage conditions encountered for real world applications. Here we show that OMV packaged PTE maintains activity over free PTE when subjected to elevated temperatures (>100-fold more activity after 14 days at 37 °C), iterative freeze-thaw cycles (3.4-fold post four-cycles), and lyophilization (43-fold). We also demonstrate how lyophilized OMV packaged PTE can be utilized as a cell free reagent for long term environmental remediation of pesticide/chemical warfare contaminated areas.

When expressing proteins and enzymes, it is often difficult to find adequate storage conditions to allow for unimpeded long-term use of a protein of interest. Even under ideal laboratory storage conditions, enzymes and proteins will often rapidly lose activity. This limitation is critical when considering production of reagents to be transported and utilized under harsh conditions. Often, the most effective enzymes are easily rendered useless through accidental exposure to non-ideal conditions. We hypothesized that packaging an enzyme within a bacterial outer membrane vesicle (OMV) would not only benefit protein production by reducing accumulation of recombinant products within the bacterium but the resulting OMV packaging would also endow the enzyme with heightened stability across a wide range of storage conditions.

Ubiquitously secreted by both Gram-negative and Gram-positive bacteria, OMVs are unilamellar proteoliposomes varying in size from 30–200 nm^1^,^2^. They serve various biological functions from cell-cell signaling, to packaging of virulence factors, and transporting of genetic material^3^,^4^,^5^,^6^. For many years, lipid-based micellar and liposomal nanoparticles have been utilized for packaging and delivering cargo^7^. Synthesis of complex liposomes, however, requires not only recombinant expression and purification of payload and targeting proteins, but also iterative synthesis and purification cycles increasing in number and cost as nanoparticle complexity increases^7^,^8^,^9^,^10^. In contrast, bacteria readily produce OMVs assembled with diverse protein and lipid compositions that naturally function similarly or better than synthetic liposomes and require only a single synthesis and purification step^11^,^12^,^13^. These properties have led to recent interest into using OMVs as therapeutic agents as evidenced through a number of studies demonstrating active targeting constructs, immunogenicity studies, and delivery of cytotoxic payloads to distinct cell populations^13^,^14^,^15^,^16^,^17^. Through engineering of genetic material, bacterial cellular machinery can be directed to produce and package recombinant products into functional OMV nanoparticles in a way that is not possible utilizing current liposomal technology^18^. This process is also much more easily scalable compared to current liposomal techniques allowing for large batch production of very complex functional reagents for use across a wide variety of applications including therapeutic, bioremediation, and industrial applications.

In a previous study, we optimized expression and packaging of a binuclear Zn/Zn organophosphate hydrolase isolated from *Brevundimonas diminuta*, phosphotriesterase (PTE, EC 3.1.8.1), into *Escherichia coli* OMVs^19^,^20^,^21^,^22^. Exposure to organophosphates is extremely dangerous as it impairs neurotransmitter function through inhibiting the hydrolysis of acetylcholine by acetylcholinesterase at neuromuscular junctions resulting in death via asphyxiation^23^. PTE was selected as an ideal enzyme for use in environmental remediation applications as it is a highly promiscuous enzyme capable of hydrolyzing a wide variety of organophosphate compounds including V and G type chemical warfare agents as well as toxic pesticides such as paraoxon, its primary substrate^24^,^25^,^26^.

We utilized a split protein SpyCatcher/SpyTag bioconjugation system to create a synthetic linkage between PTE and an abundant membrane protein, OmpA, to facilitate loading of the OMV^27^,^28^. A complete analysis of the bacterial vesiculation, packaging efficiency, overall PTE production, and enzymatic activity of the packaged PTE was carried out previously to validate the constructs^22^. Here we demonstrate how packaging PTE within an OMV utilizing these well characterized constructs greatly improves enzymatic activity across a range of conditions including: long-term storage, exposure to elevated temperatures, freeze-thaw cycles, and lyophilization allowing for enhanced potential for PTE use in real world environmental remediation applications (Fig. 1).

## Results and Discussion

### Design of the packaging construct

The exact cellular mechanism for packaging proteins into OMVs is not well understood^29^. For this reason, a biorthogonal conjugation strategy was developed to create a synthetic linkage between an enzyme of interest (PTE) and a highly expressed transmembrane porin protein (OmpA) known to be abundant in OMVs. Plasmids were designed incorporating a C-terminal fusion of SpyCatcher (~13 kDa) to PTE (~35 kDa) and SpyTag to the periplasmically facing N- or C-termini of a truncated form of OmpA (residues 22–197; ~23 kDa) (Supplementary Fig. S1)^30^,^31^. When OmpA-ST and PTE-SC are co-transformed, production of both fusion constructs facilitates isopeptide bond formation between the SpyTag and SpyCatcher which drives the packaging of the periplasmically localized PTE-SC into forming OMVs^32^. Genomic OmpA was also maintained in all of the cultures to mitigate the membrane destabilizing effects of removing the C-terminal domain of OmpA, which functions to stabilize the outer membrane through interactions with the peptidoglycan^33^,^34^,^35^. For a more detailed description of the plasmid constructs and resulting protein sequences please see the Methods section and Supplementary Fig. S2. It was shown previously that when co-transformed with PTE-SC, both the N- and C- terminal OmpA-SpyTag fusions exhibited hyper-vesiculation, improved production of PTE, and increased packaging efficiency of PTE within the OMVs. These characteristics make them desirable constructs for testing the stability of enzyme packaged OMVs as well as viable environmental remediation reagents^22^.

### Production and purification of PTE-SC loaded OMVs

*E. coli* strain BL21 (DE3) was co-transformed with both OmpA-ST and PTE-SC plasmid constructs and expanded in liquid culture in the presence of ampicillin (50 μg/ml) and chloramphenicol (25 μg/ml). PTE-SC packaged in N- and C-Terminal OmpA-ST OMVs (PTE/OMV^N^ and PTE/OMV^C^) were grown in 500 mL Terrific broth in 2L baffled culture flasks and allowed to expand for 3 h at 37 °C until an OD of 0.6–0.8 was reached. Arabinose was then added at a 0.2% final concentration initiating production of the PTE-SC. IPTG was added after an additional 3 h incubation period to a final concentration of 0.5 mM to initiate OmpA-ST production. The culture was then allowed to grow for an additional 18 h. Individual 500 mL cultures were inoculated for each of the two (N and C) OmpA-ST fusions co-transformed with PTE-SC as well as PTE-SC by itself and native PTE.

At the completion of the growth phase the intact cells and larger cellular components were removed from the culture media via centrifugation followed by 0.45 μm membrane filtration^36^. OMVs were then pelleted at ~150,000 g using an ultracentrifuge for 3 h at 4 °C. The OMV-depleted culture media was decanted and the OMV pellet was resuspended in phosphate buffered saline (PBS) at pH 7.4. It was previously verified that any free PTE-SC secreted into the culture media did not associate with the OMVs and that the centrifugal force necessary to pellet the OMVs was not sufficient to pellet free PTE-SC^22^. Free PTE-SC was purified from culture media via immobilized metal ion affinity chromatography (IMAC) utilizing the included 6xHis tag. The PTE control enzyme was expressed and purified as previously described with detailed methods included in the Supplementary Information section^37^. All samples were compared to *wt* OMVs isolated for a non-transformed *E. coli* culture grown in parallel.

### Long term storage of PTE

**Figure 2:**
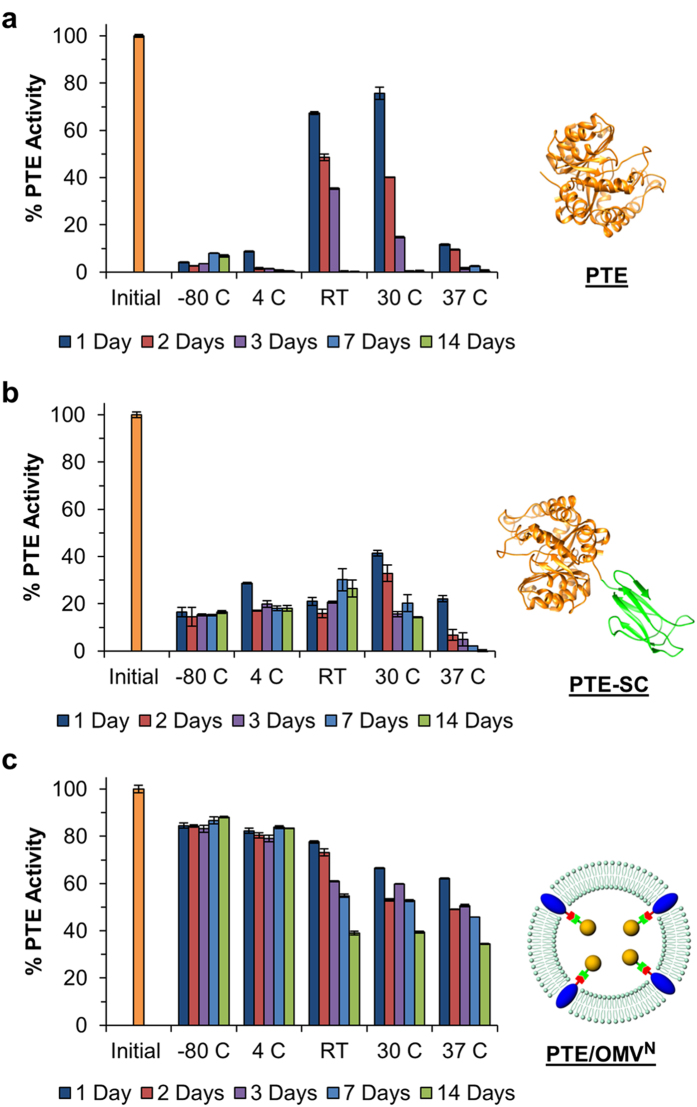
Long term and varied temperature enzyme stability. (**a**) PTE, (**b**) PTE-SpyCatcher (PTE-SC), and (**c**) N-terminal OmpA-SpyTag/PTE-SpyCatcher OMVs (PTE/OMV^N^) were assayed for PTE activity over a variety of storage conditions over the course of 14 days. PTE activity was assayed via initial velocity determination utilizing paraoxon as a substrate monitoring product formation at 405 nm. Percent activity was normalized to an initial PTE activity measurement for each sample (Day 0). Both PTE, and PTE-SC demonstrated poor stability across all storage conditions and exhibited heightened sensitivity to inactivation due to freezing and long term storage. OMV packaged PTE maintained 34.5 ± 0.2% activity after 14 days at 37 °C compared to PTE and PTE-SC which did not exhibit any activity under the same conditions. All data represents means (±one standard deviation) of triplicate experiments.

Enzyme stability was assayed over a range of storage conditions, temperatures, and time points. A stock solution of PTE (Fig. 2a), PTE-SC (Fig. 2b), PTE/OMV^N^ (Fig. 2c), and PTE/OMV^C^ was aliquoted to identical sample tubes. Tubes were placed at each storage condition (−80, 4, room temperature ~20, 30, 37 °C) and one tube was assayed immediately to determine an initial PTE activity value for comparison to each subsequent sample. Enzyme activity was evaluated by monitoring the enzyme-mediated hydrolysis of paraoxon to *p*-nitrophenol which can be measured by absorbance at 405 nm. Enzymatic progress curves for the initial samples have been included as Supplementary Fig. S3 to demonstrate the starting PTE activity for each construct tested. While low concentrations of surfactants such as Triton X-100 can be added to the sample to rupture the OMVs and improve enzyme access to substrate within the bulk solution, as demonstrated in a previous study, paraoxon freely diffuses in and out of the OMVs; therefore no additives were necessary to rupture the OMVs^22^. For future applications utilizing an alternate enzyme/substrate pair transfer of the substrate through the OMV membrane would need to be assessed. In the event substrate translocation does not occur through endogenous porin proteins these proteins may be able to be engineered to accommodate a new substrate through site-directed mutagenesis to change pore size, conformation, or charge selectivity. The potential to engineer native membrane proteins to control payload interactions with the bulk solutions significantly contrasts the capabilities of liposomes which do not possess porin proteins and therefore require the use of a surfactant to release the encapsulated enzyme for all applications.

At room temperature (RT) and 30 °C the free PTE remains fairly active over the course of the first three days but loses all activity by the end of the 14 day trial (Fig. 2a). The highest activity remaining on day 14 was present in the −80 °C storage condition retaining only 7% activity, in the absence of a cryoprotectant. It was also necessary to assay the PTE-SC fusion construct under the same storage conditions to ensure that any observed differences in retained activity associated with packaging of the PTE within an OMV was not attributed to the additional SpyCatcher domain. PTE-SC demonstrated improved stability under most assay conditions and retained ~17% activity in both the −80 °C and 4 °C storage conditions at day 14 and 26 ± 3.6% activity when stored at RT. The fusion of the more stable SpyCatcher (~13 kDa) domain to the PTE (~35 kDa) provided for slightly increased stability across nearly all storage conditions but was not sufficient to explain the level of stability observed in the OMV packaged PTE samples (Fig. 2b).

PTE/OMV^N^ exhibited greatly improved stability across all storage conditions tested. Most notable is that the N-Term packaged PTE-SC maintained 85.7 ± 2.3% activity post freezing, a 16.8- and 5.5-fold higher activity compared to PTE (5.1 ± 2.3%) and PTE-SC (15.6 ± 0.9%), respectively. OMV encapsulated PTE-SC was very stable when refrigerated at 4 °C (83 ± 0.2% activity on day 14) and exhibited exceptional stability against prolonged storage at 37 °C with 35 ± 0.2% activity remaining on day 14. PTE/OMV^C^ exhibited a nearly identical improvement to PTE stability as observed in the N-Term OMV samples (Supplementary Fig. S4). This demonstrates that the stability is not merely a result of a single linkage and orientation but rather the OMV itself is providing the supportive environment to promote resistance to inactivation of PTE over a wide range of storage conditions.

### Freeze-Thaw stability

**Figure 3:**
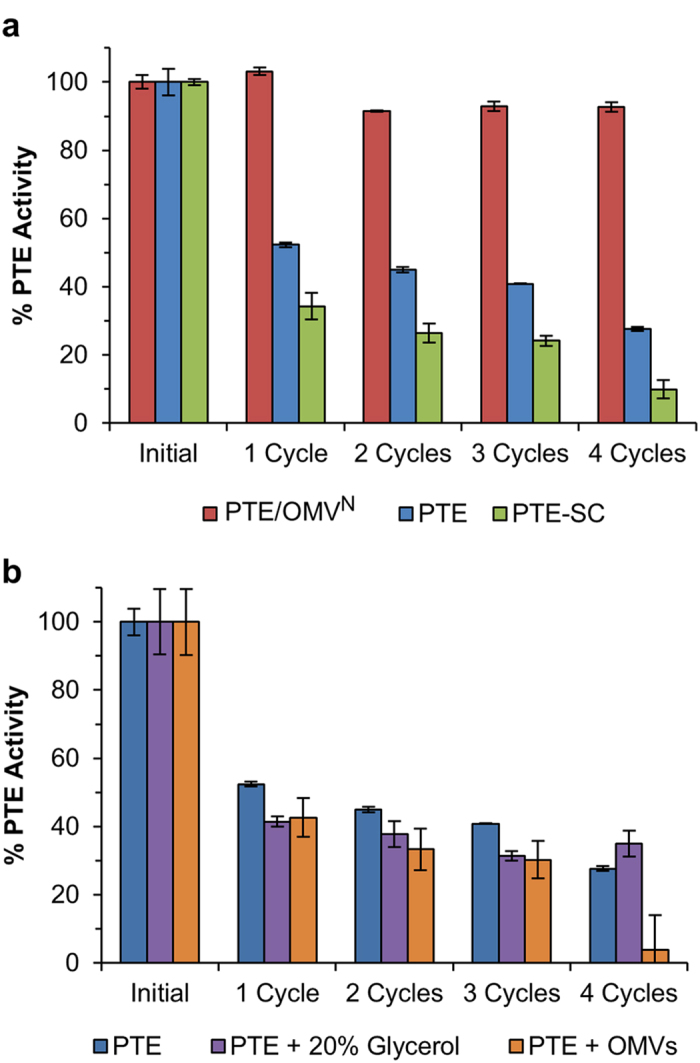
Enzyme activity post iterative freeze-thaw. (**a**) Freeze-thaw stability test of PTE/OMV^N^ compared to free PTE-SC and free PTE lacking the SpyCatcher fusion. Four cycles of freeze-thaw between −80 °C and RT were carried out and the percent PTE activity was directly compared via initial velocity measurements. Both free proteins exhibited extensive inactivation due to freeze-thaw cycles with PTE exhibiting slightly higher stability. OMV packaged PTE-SC exhibited heightened resistance to inactivation from freeze-thaw retaining 93% activity after four cycles. (**b**) PTE freeze-thaw stability was also assayed in the presence of 20% glycerol and commixed with native OMV demonstrating no improvement in resistance to inactivation due to multiple freeze-thaw cycles. All data represents means (±one standard deviation) of triplicate experiments.

Freezing is often considered to be the primary method of long-term laboratory storage for proteins and enzymes but the act of freezing itself can be a very harsh process causing inactivation. Furthermore, iterative freeze-thaw cycles are considered to be one of the most detrimental processes that an enzyme can be subjected to making it an ideal assay to compare the stability of free PTE to OMV encapsulated PTE. Stock samples were aliquoted in PBS pH 7.4 and snap frozen in liquid nitrogen for a total of four cycles of freeze-thaw storing the samples at −80 °C and RT for 1 h incubation periods. PTE activity was directly compared to initial velocity measurements of the same sample not subjected to any freeze-thaw cycles. No cryoprotectants were included in this assay to demonstrate the worst case scenario for accidental freeze-thaw of a sample that may occur during transportation or improper storage. Both PTE and PTE-SC exhibited extensive inactivation due to freeze-thaw cycles with PTE exhibiting slightly higher stability retaining 28% activity after four cycles compared to PTE-SC retaining only 10% activity (Fig. 3a). OMV packaged PTE-SC exhibited heightened resistance to inactivation from freeze-thaw retaining 93% activity after four cycles. This demonstrates a 9.3- and 3.3-fold increase in active PTE remaining after four freeze-thaw cycles in the OMV encapsulated PTE-SC construct compared to naked PTE-SC and PTE, respectively. This result was consistent with the slightly more freeze-thaw sensitive PTE/OMV^C^ construct retaining 68% activity after four cycles (Supplementary Fig. S5).

We sought to also assay the PTE activity utilizing ‘good laboratory practices’ by adding glycerol (20% final concentration) to the buffer prior to freezing the samples. Glycerol is a common cryoprotectant that is known to help protect proteins from inactivation resulting from freeze-thaw by inhibiting the formation of ice crystals upon freezing^38^. In addition, non-encapsulated free PTE was also assayed in the presence of native OMVs to ensure that association to the exterior surface of the OMVs would not have a similar effect in improving freeze-thaw stability of the enzyme. OMVs were isolated from *wt E. coli* via ultracentrifugation and were spiked into the free PTE sample prior to freezing at the same concentration as was present in the PTE/OMV^N/C^ samples above. After four cycles of freeze-thaw neither the addition of 20% glycerol nor the OMVs improved the free PTE stability when exposed to multiple freeze-thaw cycles (Fig. 3b). This assay also helps to demonstrate that the PTE must be packaged within the OMVs to enhance stability and cannot simply be added to the solution prior to freezing. While it was surprising that the addition of glycerol did not help enzyme stability, glycerol is only a best practice technique and does not always improve protein stability.

### Lyophilization

**Figure 4:**
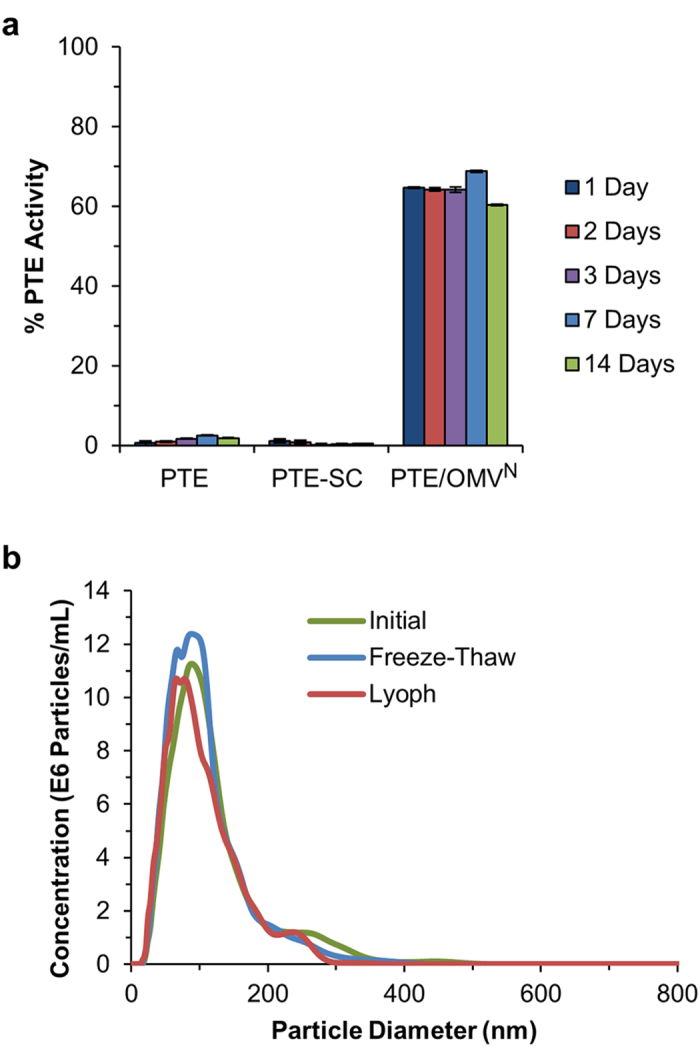
OMV stability to lyophilization and rehydration. (**a**) PTE, PTE-SC, and PTE/OMV^N^ were assayed for stability post lyophilization in PBS pH 7.4 with no protectants. Lyophilized samples were stored at RT for 14 days. Non-OMV encapsulated PTE did not survive the lyophilization process compared to a >60% retained activity by OMV encapsulated PTE-SC. (**b**) OMV size distribution and recovery post freeze-thaw and lyophilization were assessed on a NanoSight LM10 particle tracking system. The OMVs retained both their size distribution and absolute particle number post rehydration. All data represents means (±one standard deviation) of triplicate experiments.

Lyophilization (aka freeze-drying) is the process by which water is removed from a frozen sample via sublimation, in which solid water is removed as a gas without going through the liquid phase. Lyophilization is considered to be the gold standard for ultra-long storage of a reagent as it requires less strict regulation over the temperatures a reagent is stored at and also significantly reduces the weight of a reagent facilitating easier transportation^39^,^40^,^41^. To assess PTE stability to lyophilization stock samples were aliquoted in PBS pH 7.4, snap frozen in liquid nitrogen, and lyophilized overnight to ensure that all water was removed from the samples. Lyophilized samples were stored at RT and then rehydrated on days 1, 2, 3, 7, and 14 to assess enzyme activity. Both PTE and PTE-SC were dramatically affected by this process retaining only 1.5 and 0.6% activity, respectively (Fig. 4a). This is in contrast to the PTE/OMV^N^ construct which retained >60% activity upon lyophilization and the PTE/OMV^C^ which retained >70% activity (Supplementary Fig. S6). OMV packaged PTE-SC, therefore, demonstrated a minimum improvement in retained PTE activity of 43-fold. Passively packaged PTE encapsulated into OMV, albeit at a much lower loading efficiency and greatly reduced overall PTE yield^22^, exhibited similarly retained stability post freeze-thaw and lyophilization demonstrating that surface immobilization to the interior wall is not necessary to attain the stability enhancing benefits of OMV packaging (Supplementary Fig. S7).

To explore this phenomenon further the OMV size distribution and concentration were assessed post freeze-thaw and post lyophilization-rehydration using NanoSight NTA 2.3 Nanoparticle Tracking and Analysis software. Ultracentrifuge concentrated vesicles were diluted 2,000-fold in PBS pH 7.4 and particle tracking was carried out at RT via analysis of 90 s video clips. Following lyophilization and rehydration, PTE/OMV^N^ demonstrated excellent resistance to rupture and aggregation as demonstrated by nearly identical size distribution and calculated concentration (Fig. 4b). This is likely a result of the transmembrane porin proteins present in the OMV membrane allowing for the transfer of small molecules and water in and out of the OMV. Synthetic liposomes often require additives such as glycerol or sucrose prior to freezing to prevent rupture and aggregation post thaw because they lack porin proteins and therefore have no built in mechanism to mitigate pressure variations^42^. PTE/OMV^C^ also exhibited resistance to rupture and aggregation upon freeze-thaw but their size distribution was more sensitive to the lyophilization process (Supplementary Fig. S8).

### Long-term remediation

**Figure 5:**
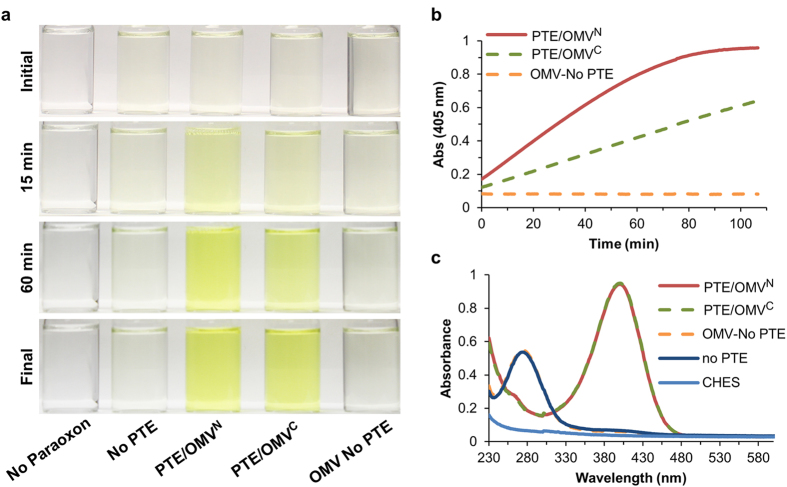
Proof of concept paraoxon remediation. Larger-scale remediation of paraoxon utilizing lyophilized PTE/OMV^N^ and PTE/OMV^C^. (**a**) Lyophilized OMV powder was added directly to paraoxon contaminated water in scintillation vials and pictures were taken over a 3 h period of time. (**b**) Paraoxon degradation was monitored at 405 nm demonstrating complete remediation within 90 and 180 min for the PTE/OMV^N^ and PTE/OMV^C^, respectively. (**c**) Absorbance spectrums were taken to demonstrate complete conversion of paraoxon (280 nm) to *p*-nitrophenol (405 nm). Despite differing amounts of initial PTE, complete remediation of paraoxon was observed by both OMV constructs.

OMVs are essentially a fully biodegradable vehicle that is environmentally inert. Unlike many chemical catalysts which must be removed or processed post-remediation, OMVs can be directly reabsorbed into the environment. This makes enzyme-filled OMVs an ideal reagent for bioremediation at the point-of-concern. To demonstrate this potential, lyophilized PTE-SC OMVs were employed as a reagent for the remediation of 20 mL samples of paraoxon contaminated water. Concentrated N- and C-Term packaged PTE-SC OMVs were lyophilized and the powder was directly added to the contaminated water. Controls were also assayed to account for any endogenous paraoxon degradation as well as potential nonspecific cleavage by native OMVs in the absence of PTE. The vials were mixed briefly and placed at RT unstirred for the remainder of the remediation (Fig. 5a). Reaction progress was monitored following the conversion of paraoxon by measuring the absorbance at 405 nm (Fig. 5b). There was negligible breakdown of paraoxon in the absence of PTE or in the presence of native OMVs. The rate of conversion differed between the PTE/OMV^N^ and PTE/OMV^C^ samples due to differing amounts of packaged PTE, as evidenced by a shallower initial velocity observed in the PTE/OMV^C^ sample. Absorbance spectrums for each sample were taken four hours after the start of the experiment displaying uncatalyzed paraoxon and hydrolyzed paraoxon at 280 and 405 nm absorbance maximums, respectively (Fig. 5c). Despite the differing initial velocities of the two OMV samples both resulted in complete conversion of paraoxon. Lyophilized free PTE demonstrated minimal conversion of paraoxon as it retained only ~1% activity post lyophilization (Supplementary Fig. S9). This experiment was conducted as such to demonstrate that packaged PTE-SC can be delivered directly as a lyophilized powder and despite varying initial concentrations of enzyme complete conversion of the contaminant can be attained.

**Figure 6:**
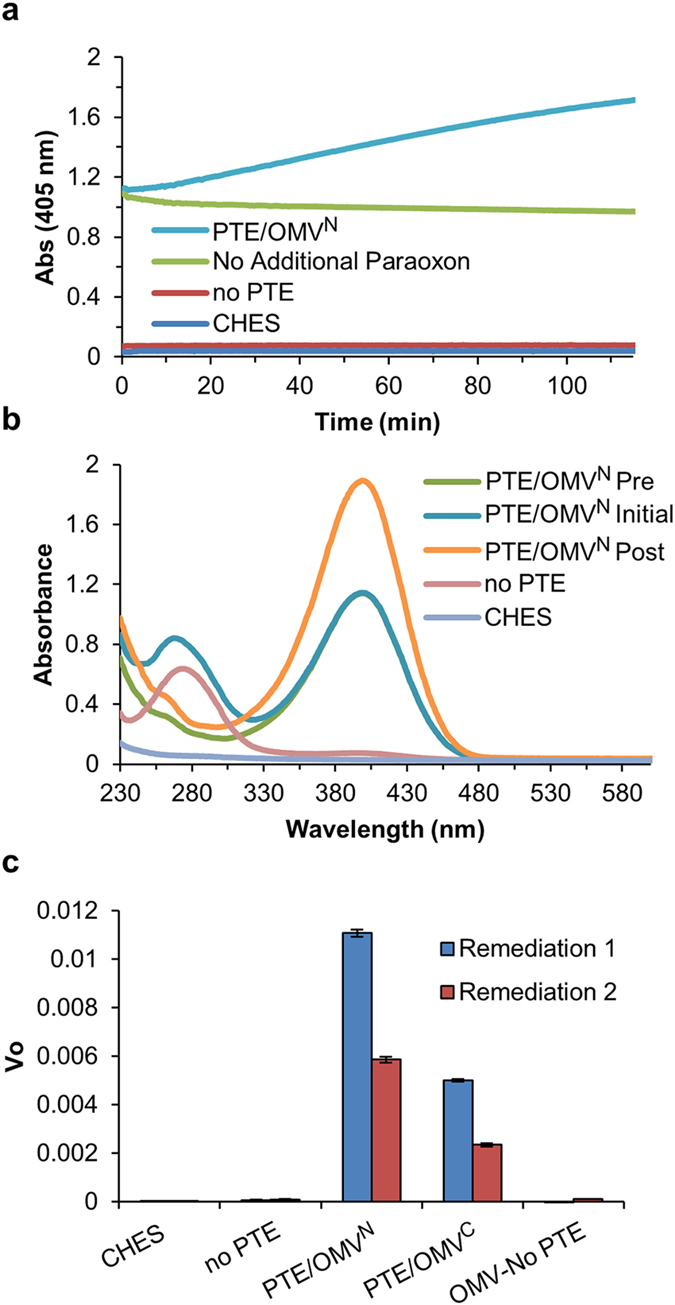
Long-term paraoxon remediation. After 4 days at RT storage a second remediation cycle utilizing the exact same samples from Fig. 5 were carried out with an equal quantity of paraoxon. (**a**) Progress curves were monitored for paraoxon degradation at 405 nm with complete remediation utilizing the PTE/OMV^N^ within 180 min. (**b**) Absorbance spectrums were taken after 4 h to demonstrate complete conversion of paraoxon (280 nm) to *p*-nitrophenol (405 nm). [PTE/OMV^N^ Pre: spectrum prior to the addition of the second bolus of paraoxon, PTE/OMV^N^ Initial: spectrum directly following the addition of the second bolus of paraoxon, PTE/OMV^N^ Post: spectrum taken at the end of the remediation]. (**c**) Despite differing amounts of initial PTE, both PTE/OMV^N^ and PTE/OMV^C^ were still capable of complete remediation of the paraoxon with 53 and 47% retained activity, respectively. All data represents means (±one standard deviation) of triplicate experiments.

It is important to ensure that there is sufficient active PTE present to remediate paraoxon contamination completely in the case of very high concentrations of paraoxon or a repeat contamination of the region. To demonstrate how PTE-SC packaged OMVs perform under such a circumstance the identical samples from the initial 20 mL remediation above were stored at RT for four days and were then challenged with an additional bolus of paraoxon at the same concentration as the initial paraoxon challenge. The conversion of paraoxon was again monitored at 405 nm (Fig. 6a) and an absorbance spectrum was taken 8 h after the start of the experiment demonstrating complete conversion of the paraoxon (Fig. 6b). The PTE/OMV^C^ remediation data is presented separately in the interest of clarity and can be found in Supplementary Fig. S10. There was no breakdown of paraoxon in the absence of PTE or in the absence of an additional bolus of paraoxon. The rate of conversion again differed between the PTE/OMV^N^ and PTE/OMV^C^ samples but was consistent with the stability measurements for OMV encapsulated PTE-SC after 4 days of RT storage exhibiting 53 and 48% retained activity based on initial velocity measurements, respectively (Fig. 6c). This experiment demonstrates that packaged PTE-SC retains significant activity despite lyophilization, over many days of RT storage in the presence of paraoxon breakdown products, and despite varying initial concentrations of enzyme and still provides for complete remediation of the paraoxon contaminated sample. It is important to note that this type of remediation is scalable in that more enzyme provides for faster remediation and longer periods of time allow for more overall paraoxon to be degraded.

## Conclusions

In summary, we have demonstrated that through a synthetic biology approach a highly evolved bacterial vehicle, such as an outer membrane vesicle, can be programed to package and enhance stability of enzymes of interest under a diverse set of storage conditions. Through packaging the PTE within OMVs the enzyme is much less susceptible to inactivation via freezing or lyophilization making this functional material a powerful and robust reagent for improved implementation under harsh conditions compared to free enzyme. The enhanced stability observed not only allows for prolonged storage of the active enzyme prior to use but also ensures that enzyme remains active over long periods of contaminant digestion. While it is unclear as to the exact mechanism that contributes to the improved stability of the OMV encapsulated PTE-SC we hypothesize that the diverse nature of the protein, lipid, and polysaccharide components that comprise the OMV affords the packaged enzymes access to numerous microenvironments to support stability over a wide range of conditions. Proteomic analysis of OMV samples also indicate the presence of periplasmic chaperone proteins that are passively packaged within OMVs which may also be contributing to the enhanced enzyme stability^28^,^29^. Packaging of recombinant proteins in OMVs combines all of the benefits of evolutionarily optimized nanoparticles wholly produced by bacteria for the cell-free degradation of chemical contaminants without fear of releasing mutant bacterial strains into the environment. While PTE was selected for this unique application the results of this study can be used to design analogous protein packaging strategies for use in diverse pharmaceutical delivery, environmental remediation, and industrial applications.

## Methods

### Construction of *E. coli* expression plasmids

Genes encoding for a truncated OmpA (amino acids 22–197) with the SpyTag sequence appended to either the N-terminus or C-terminus were synthesized by GenScript (Piscataway, NJ) in a pUC57 shuttle vector with flanking NcoI and NotI restriction sites. The SpyTag in each construct was flanked by a spacer amino acid sequence (GGGS). Synthesized plasmids were digested with NcoI-HF and NotI-HF (New England Biolabs, Ipswich, MA) and cloned into identical sites in the pET22b expression vector (Novagen, Billerica, MA). A second expression vector utilizing a compatible origin of replication was constructed for the co-expression of the PTE-SC utilizing a modified pACYC184 vector (New England Biolabs) under arabinose control containing: a p15a origin, twin-arginine translocation substrate TMAO reductase (TorA), a hexa-histidine sequence, and several unique restriction endonuclease cleavage sites (vector referred to as pACYC184 AraC). The PTE and SpyCatcher genes were combined through a series of PCR amplification, restriction digest reactions and ligations. Both the pMinit PTE-SC and pACYC184 AraC were digested with XhoI, gel purified, and the relevant fragments ligated using T4 DNA ligase. Free-PTE plasmid preparation, expression and purification as well as protein sequences for each construct can be found in the supplementary information section.

### Bacterial growth conditions

*E. coli* strain BL21 (DE3) containing the pET22 OmpA-ST (C/N) and pACYC184 AraC PTE-SC plasmids were maintained on solid medium and expanded in overnight cultures in the presence of ampicillin (100 μg/mL) and chloramphenicol (25 μg/mL). For OMV production, 5 mL of overnight culture was used to inoculate 500 mL of TB in baffled culture flasks. The resulting culture was allowed to grow for 3 h at 37 °C until an OD_600_ of 0.6–0.8 was reached. Arabinose was added to a 0.2% final concentration. After an additional 3 h incubation IPTG was added to a final concentration of 0.5 mM and the culture was allowed to grow for 18 h at 37 °C.

### OMV purification

All conditioned bacterial culture media was centrifuged two times at 7,000 g for 15 min at 4 °C and then 0.45 μm membrane filtered. This removed all intact bacteria and undesired large cellular material. OMVs (36 mL of culture media) were then pelleted in Ultra-Clear (25 × 89 mm) centrifuge tubes purchased from Beckman Coulter (Brea, CA) at 29,000 RPM (~150,000 g) in a Sorvall WX Ultra 90 centrifuge using an AH-629 rotor for 3 h at 4 °C. The OMV depleted culture media was decanted and the OMV pellet was resuspended in 1 mL of PBS pH 7.4.

### PTE kinetic assays

All enzymatic assays were conducted in 2-(Cyclohexylamino)ethanesulfonic acid (CHES) buffer (150 mM, pH 8) at 25 °C. Enzyme-mediated hydrolysis of the paraoxon substrate (1:1,000 dilution) purchased from Chem Service (West Chester, PA) to *p*-nitrophenol was monitored at 405 nm. Initial velocities were determined by the slope of the first 5 min of reaction and were utilized to compare the relative quantity of active PTE in each sample to determine PTE stability under all storage conditions tested.

### NanoSight

Vesicle size distribution and quantitation were performed on a NanoSight LM10 system (Salisbury, UK) using NTA 2.3 Nanoparticle Tracking and Analysis software. Samples were diluted 2,000-fold in PBS pH 7.4 with camera shutter (13.8 ms) and gain (324) optimized to enhance data collection. Videos (90 sec) were taken and frame sequences were analyzed under auto particle detection and tracking parameters: detection threshold, pixel blur, min track length, and min expected particle size. All samples were run at RT and allowed to equilibrate 15 min prior to analysis.

### Freeze-thaw stability testing

OMV encapuslated PTE-SC was compared to free PTE-SC and free PTE for stability against multiple cycles of freeze-thaw. The samples were assayed for initial PTE activity via initial velocity measurements. The samples were then snap frozen in liquid nitrogen and placed at −80 °C for 1 h and were then allowed to warm to RT for 1 h for a total of four freeze-thaw cycles. PTE activity was assayed at the completion of each thaw to assess enzyme stability.

### Long-term rememdiation

Paraoxon, 1 mL of 1:1,000 dilution, was added to 19 mL of CHES buffer pH 8.0. Ultracentrifuge concentrated OMVs (36-fold) purified from culture supernatant were diluted to 1 mL in PBS pH 7.4 and lyophilized overnight. Lyophilized powder was directly added to the 20 mL reaction vial. A sample was taken from the vial after an initial 10 sec mixing and was used to track the progress of the reaction at 405 nm. Pictures were taken every 15 min and no further mixing occurred. At the completion of the reaction the samples were stored at RT for 4 days and then spiked with an additional 1 mL of 1:1,000 diluted paraoxon. The progress was again tracked and an absorbance spectrum at the completion of paraoxon remediation was performed.

## Additional Information

**How to cite this article**: Alves, N. J. *et al.* Protecting enzymatic function through directed packaging into bacterial outer membrane vesicles. *Sci. Rep.* **6**, 24866; doi: 10.1038/srep24866 (2016).

## Supplementary Material

Supplementary Information

## Figures and Tables

**Figure 1:**
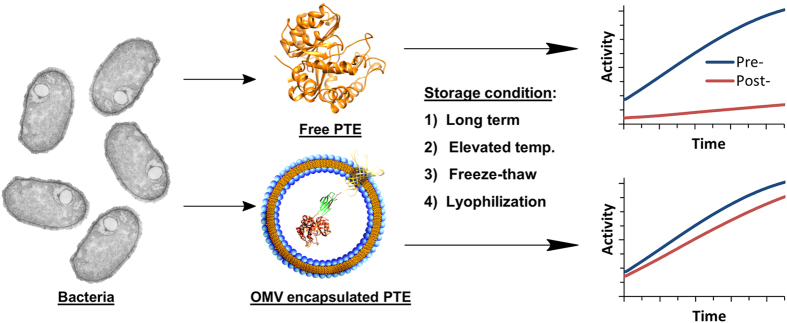
PTE packaging and stability schematic. Pictured above is a schematic representation of bacterial produced free-PTE and isopeptide bonded N-terminal OmpA-SpyTag/PTE-SpyCatcher packaged within an outer membrane vesicle (OMV). The fusion of PTE to the transmembrane porin protein (OmpA) through the SpyTag/SpyCatcher construct facilitates incorporation of the PTE within the OMV. The OMV provides a protective environment that reduces inactivation of the encapsulated enzyme under nearly all storage conditions resulting in prolonged shelf-life and enhanced stability.
