# Supplementary material for: Protecting enzymatic function through directed packaging into bacterial outer membrane vesicles
Source: Sci Rep. 2016 Apr 27;6:24866. doi: 10.1038/srep24866 (PMC4846811; doi:10.1038/srep24866)
Supplement: Supplementary Information [file srep24866-s1.pdf]

# Supporting Information:

# *Protecting enzymatic function through directed packaging into bacterial outer membrane vesicles*

*Nathan J. Alves[1], Kendrick B. Turner[2], Igor L. Medintz[2], Scott A. Walper*[2]*

[1]*National Research Council*

500 Fifth Street NW (Keck 576)

Washington, DC 20001

[2]*Center for Bio/Molecular Science & Engineering*

U.S. Naval Research Laboratory

Washington, DC 20375, USA

*Scott A. Walper
Research Biologist
Center for Bio/Molecular Science & Engineering
Naval Research Laboratory (NRL)
4555 Overlook Ave., SW
Washington, DC 20375
Voice: 1-202-404-6070
Email: Scott.Walper@nrl.navy.mil

**<u>Table of Contents</u>:**

**Additional Methods. Free-PTE plasmid preparation, expression and purification**

**Figure S1. Schematic representation of N-Term OmpA-ST, C-Term OmpA-ST, PTE-SC**

**Figure S2. Protein sequences: N-Term OmpA-ST, C-Term OmpA-ST, PTE-SC**

**Figure S3: Initial progress curves demonstrating starting PTE activity for each construct**

**Figure S4. PTE/OMV$^C$ long term and varied temperature enzyme stability**

**Figure S5. PTE/OMV$^C$ enzyme activity post iterative freeze-thaw**

**Figure S6. PTE/OMV$^C$ packaged PTE stability to lyophilization and rehydration**

**Figure S7. Passively packaged PTE stability to freeze-thaw and lyophilization**

**Figure S8. PTE/OMV$^C$ stability to lyophilization and rehydration NanoSight**

**Figure S9. Lyophilized free PTE long-term paraoxon remediation**

**Figure S10. PTE/OMV$^C$ long-term paraoxon remediation**

**Additional Methods:**

**Free-PTE plasmid preparation, expression and purification**

Restriction enzymes and T4 DNA ligase were obtained from New England Biolabs (Ipswich, MA). Unless otherwise stated, chemical were obtained from either Sigma Aldrich or Fisher Scientific. Chemically competent TOP10 cells (Life Technologies) were used for routine cloning and *E. coli* BL21(DE3) cells (New England Biolabs) were used for protein expression.

The gene for the phosphotriesterase (PTE (EC 3.1.8.1)) from *Brevundimonas diminuta* (previously *Pseudomonas diminuta*) was synthesized without the native leader sequence by Genscript. To facilitate cloning into either periplasmic (pET22) or cytoplasmic expression vectors (pET28) the gene was synthesized with flanking *NcoI* (5' end) and *XhoI* (3' end). The addition of the restriction endonuclease sites resulted in the addition of two amino acids at either termini; Met-Gly at the N-terminus and Leu-Glu at the C-terminus preceding the hexa-histidine epitope tag. The PTE gene was excised from the pUC57 plasmid vector provide by Genscript via restriction enzyme digest and purified from an agarose gel using the QIAgen MinElute Gel Extraction kit. The bacterial expression vectors (pET22 and pET28) were similarly digested and purified. Ligation was conducted overnight at 16°C at a 3:1 insert to vector ratio. Following transformation and selection, plasmid DNA from individual clones was sent to Eurofins Operon for sequencing. Plasmid DNA from positive clones was transformed to BL21(DE3) cells for protein expression.

Expression was conducted in 500 ml of Terrific Broth supplemented with kanamycin (25 μg/ml). Cultures were grown to mid-log stage at 37°C then induced with isopropyl-ß-D-1-thiogalactopyranoside (0.5 mM final concentration) for 15 hours at 30°C. Cells were collected via centrifugation then stored at -80°C overnight. Cell pellets (7-8 g average wet cell weight) were resuspended in 30 ml of lysis buffer (50 mM phosphate (pH 7.4), 1 mM EDTA, 0.01% Triton X-100, 1mg/ml lysozyme) and incubated on ice with agitation for 30 minutes. Cells were ruptured using a Branson sonifier. Soluble material was separated via centrifugation and transferred to a 50 ml conical tube. The PTE was incubated with NiNTA resin (GE Healthcare) for 2 hours at 4°C with constant rotation on a Dynal Rotisserie. Batch immobilization and subsequent IMAC purification was performed in wash buffer (50mM phosphate buffer (pH 7), 0.4 M NaCl, 25 mM imidazole, 0.05% Tween-20). Following the incubation period the resin was batch washed 3 times with 30x bed volume for each wash. Protein was eluted with wash buffer containing 0.3 M imidazole. Eluates were immediately loaded to an Enrich SEC650 FPLC column and further purified using the BioRad Biologic System.

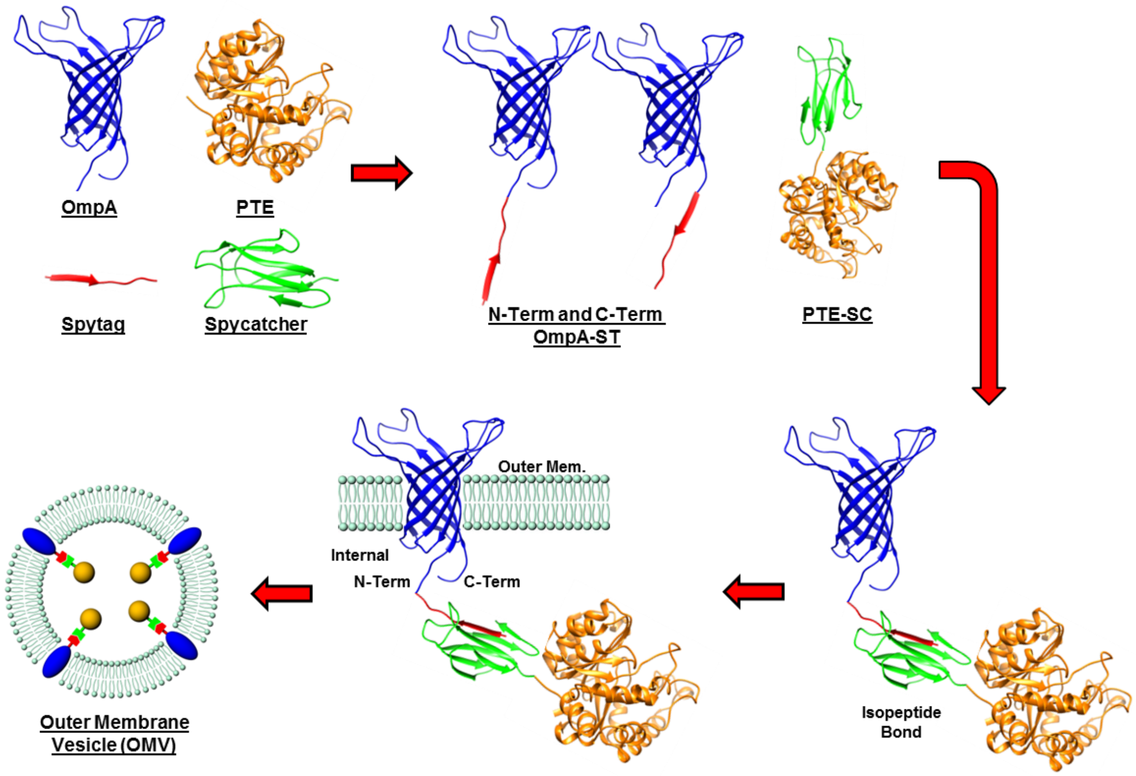


**Figure S1. Schematic representation of N-Term OmpA-ST, C-Term OmpA-ST, PTE-SC**. Crystal structures for the proteins utilized in the biorthogonal membrane conjugation of PTE for packaging into outer membrane vesicles: OmpA, PTE, SpyTag and SpyCatcher (PDB: 2GE4, 1PTA, 4MLI, 4MLI, respectively). Two separate OmpA-SpyTag fusion constructs were synthesized: C-terminal (C) and N-terminal (N) OmpA loop fusions. Pictured above is a schematic representation of each fusion component and N-terminal OmpA-SpyTag and PTE-SpyCatcher forming an isopeptide bond at the bacterial outer membrane. This membrane fusion facilitates incorporation of the PTE within the OMVs that are released from the bacteria surface.

**a)** *OmpASpyN*: OmpA leader sequence in cyan, SypTag sequence in gray, OmpA sequence green.

1 MKKTAIAIAV ALAGFATVAQ AGGGSAHIVM VDAYKPTKGG GSAPKDNTWY TGAKLGWSQY
61 HDTGFINNNG PTHENQLGAG AFGGYQVNPY VGFEMGYDWL GRMPYKGSHH HVENGAYKAQ
121 GVQLTAKLGY PITASDDLDI YTRLGGMVWR ADTKSNVYGK NHDTGVSPVF AGGVEYAITP EIATRLEYQW
191 TNNIGDAHTI GTRPDNGMLS LGVSYRFG

***b) OmpASpyC*:**

1 MKKTAIAIAV ALAGFATVAQ AAPKDNTWYT GAKLGWSQYH DTGFINNNGP THENQLGAGA
61 FGGYQVNPYV GFEMGYDWLG RMPYKGSHHH VENGAYKAQG VQLTAKLGYP ITASDDLDIY
121 TRLGGMVWRA DTKSNVYGKN HDTGVSPVFA GGVEYAITPE IATRLEYQWT NNIGDAHTIG
181 TRPDNGMLSL GVSYRFGGGG SAHIVMVDAY KPTKGGGS

***c) PTE-SC with TorA leader:*** leader in cyan, SpyCatcher in magenta, PTE in Yellow.

1 MNNNDLFQAS RRRFLAQLGG LTVAGMLGPS LLTPRRATAA QARGSHHHHH HLEGTSIGTGD
61 RINTVRGPITI SEAGFTLTHE HICGSSAGFLR AWPEFFGSRK ALAEKAVRGL RRARAAGVRT IVDVSTFDIG
121 RDVSLLAEVS RAADVHIVAA TGLWFDPPLS MRLRSVEELT QFFLREIQYG IEDTGIRAGI IKVATTGKAT
181 PFQELVLKAA ARASLATGVP VTTHTAASQR DGEQQAAIFE SEGLSPSRVC IGHSDDTDDL SYLTALAARG
241 YLIGLDHIPH SAIGLEDNAS ASALLGIRSW QTRALLIKAL IDQGYMKQIL VSNDWLFGFS SYVTNIMDVM
301 DRVNPDGMAF IPLRVIPFLR EKGVPQETLA GITVTNPARF LSPTLRASGT GGSVDTLSGL SSEQGQSGDM
361 TIEEDSATHI KFSKRDEDGKE LAGATMELRD SSGKTISTWI SDGQVKDFYL YPGKYTFVET AAPDGYEVAT
421 AITFTVNEQG QVTVNGKATK GDAHISGGGG ELVD*

**Figure S2: Protein Sequences. a)** OmpA-N-ST, **b)** OmpA-C-ST, **c)** PTE-SC.

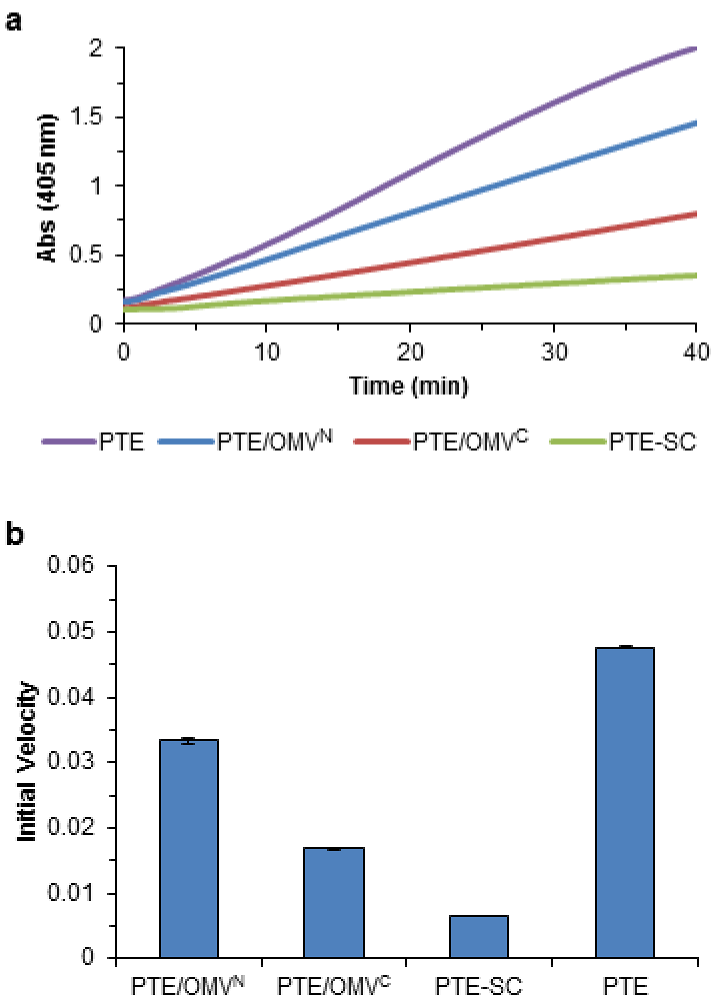


**Figure S3: Progress curves and initial velocity determination for stability experiments.** a) While each sample was internally compared against its own initial activity sample for the purposes of the long term storage, freeze-thaw and lyophilization assays it is also important to compare initial activity measurements between each construct tested. Here we show the progress curves for the initial samples tested under identical assay conditions. b) We previously showed that all constructs tested exhibited a similar $k_{cat}$ (~2,200 $s^{-1}$) allowing us to therefore use initial velocity measurements to provide for an approximate determination of active PTE concentration in each sample. We based our calculations on the known concentration of free PTE determined by absorbance at 280 nm in conjunction with a calculated extinction coefficient. Direct quantification of the OMV encapsulated PTE is difficult due to the diverse protein make-up of the sample as well as the high lipid content making many protein quantification techniques result in inaccurate measurements. The initial velocities are calculated as the slope of the linear portion of the progress curves from panel (a). It is important to note that PTE is a very active enzyme and therefore these assays were conducted at dilute concentrations of PTE which also mitigates any potential risk of protein aggregation that may adversely affect enzyme activity. The calculated concentration of PTE in the PTE/OMV$^{N}$, PTE/OMV$^{C}$, PTE-SC, and PTE samples were 2.4, 1.2, 0.5, and 3.4 nM, respectively. This demonstrates an approximate 3-fold variation

between PTE/OMV$^N$, PTE/OMV$^C$, and PTE samples and a <7-fold PTE concentration difference between PTE-SC compared to the highest concentration of free PTE.

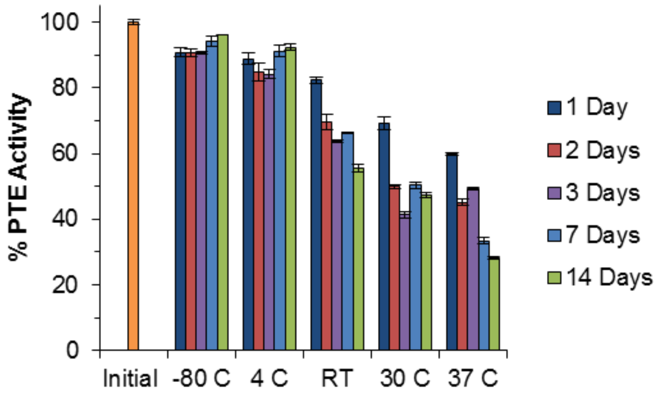


**Figure S4: Long term and varied temperature enzyme stability.** PTE-SC packaged within C-terminal OmpA-ST OMVs (PTE/OMV$^C$) exhibited greatly improved stability across all storage conditions. OMV C-Term packaged PTE-SC retained nearly 6-fold PTE activity when stored at -80°C on day 14 compared to free PTE-SC. Packaging within the OMV considerably stabilizes the PTE across a wide range of temperatures (-80°C to 37°C). Both C-Term and N-Term OMV packaged PTE-SC constructs demonstrated greatly improved PTE stability across all storage conditions allowing PTE to potentially be used in ways that were not previously thought possible.

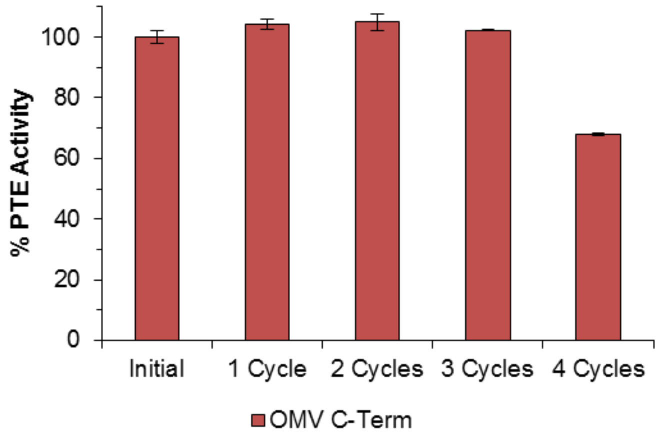


**Figure S5: Enzyme activity post iterative freeze-thaw.** Freeze-thaw stability test of PTE/OMV$^C$. Four cycles of freeze-thaw between -80°C and room temperature were carried out and the percent PTE activity was directly compared via initial velocity measurements. OMV packaged PTE-SC exhibited heightened resistance to inactivation from freeze-thaw retaining nearly 100% activity after three cycles and 68% activity after four cycles.

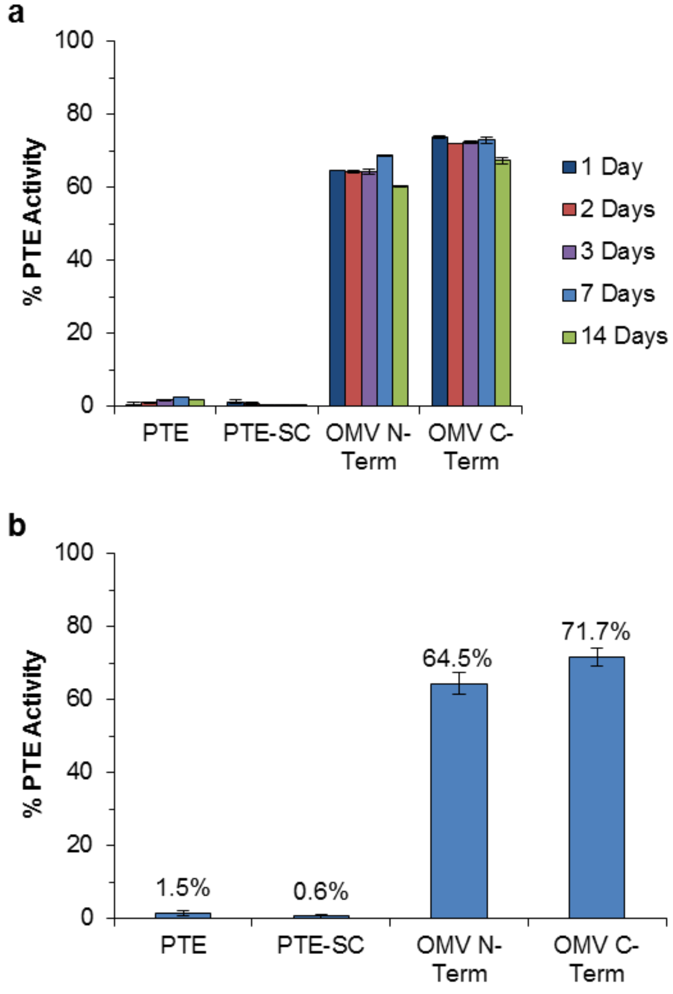


**Figure S6: OMV packaged PTE stability to lyophilization and rehydration.** **a)** PTE, PTE-SC, PTE/OMV$^N$ and PTE/OMV$^C$ were assayed for stability post lyophilization in PBS pH 7.4 with no additives. Lyophilized samples were stored at RT for 14 days. Non-OMV encapsulated PTE did not survive the lyophilization process compared to a >60% retained activity by PTE/OMV$^N$ and >70% retained activity by PTE/OMV$^C$. **b)** Average of all five lyophilized samples for each of the assayed constructs comparing free enzyme to packaged enzyme activity upon rehydration. PTE-SC packaged within OMVs demonstrated a minimum improvement in retained PTE activity of 43-fold.

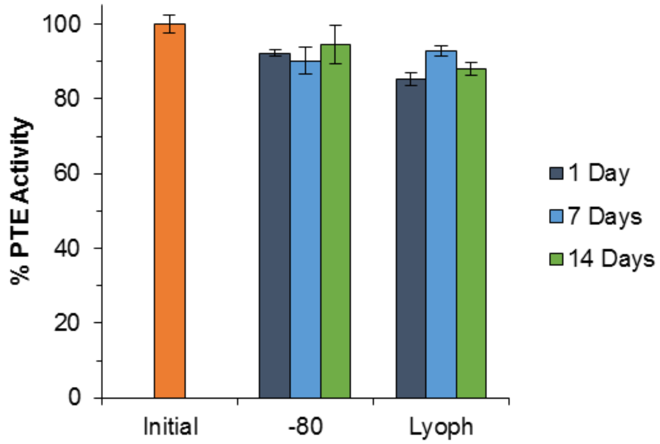


**Figure S7: Passively packaged PTE stability to freeze-thaw and lyophilization.** When PTE is expressed in *E. coli* and targeted to the periplasm there is a small amount that passively gets packaged within OMVs. Here we show the stability of passively packaged PTE within OMVs to freeze-thaw and lyophilization. The passively packaged PTE exhibits greatly enhanced stability when compared to free PTE and even exhibits slightly improved stability over both the PTE/OMV$^{N}$ and PTE/OMV$^{C}$ constructs. This demonstrates that the PTE does not need to be tethered to the inner wall of the OMV through the SpyTag/SpyCatcher linkage and rather passively encapsulated enzyme, or enzymes packaged within OMV via alternate means, will also benefit from the stability enhancing features provided by the shelter of the OMV. The primary purpose for the inclusion of the Spytag and SpyCatcher fusions is to improve overall production of PTE and enhance packaging efficiency within the OMVs by driving the linkage of PTE to OmpA. The overall expression of PTE without co-transforming PTE-SC and mutant OmpA-ST is 24-fold less with 13-fold less OMV packaged within OMVs compared to the PTE/OMV$^{C}$ construct. There is also a marked decrease (3-fold) in overall vesiculation in the absence of mutant OmpA-ST.

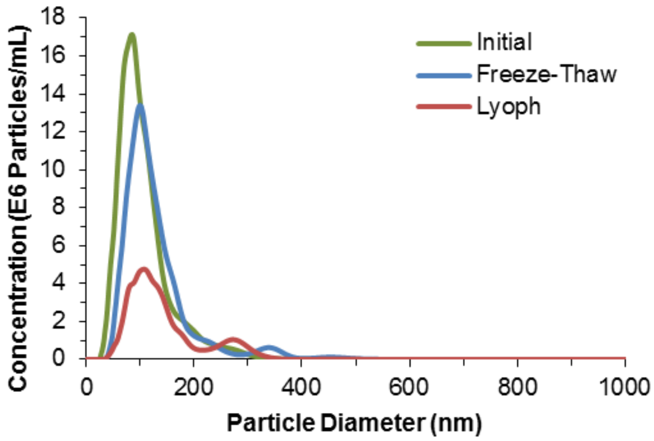


**Figure S8: OMV stability to lyophilization and rehydration.** NanoSight size distribution and OMV recovery post freeze-thaw and post lyophilization and rehydration were assessed on a NanoSight LM10 particle tracking system using NTA 2.3 Nanoparticle Tracking and Analysis software. UC concentrated vesicles were diluted 2,000-fold in PBS pH 7.4 and particle tracking was carried out at room temperature via analysis of 90 s video clips. PTE/OMV$^{C}$ demonstrated excellent resistance to rupture upon freeze-thaw but was not as stable to lyophilization in maintaining membrane stability. Despite the observed aggregation this had little impact on the PTE activity upon rehydration as indicated previously by the initial velocity determination in Supplementary Fig. 5.

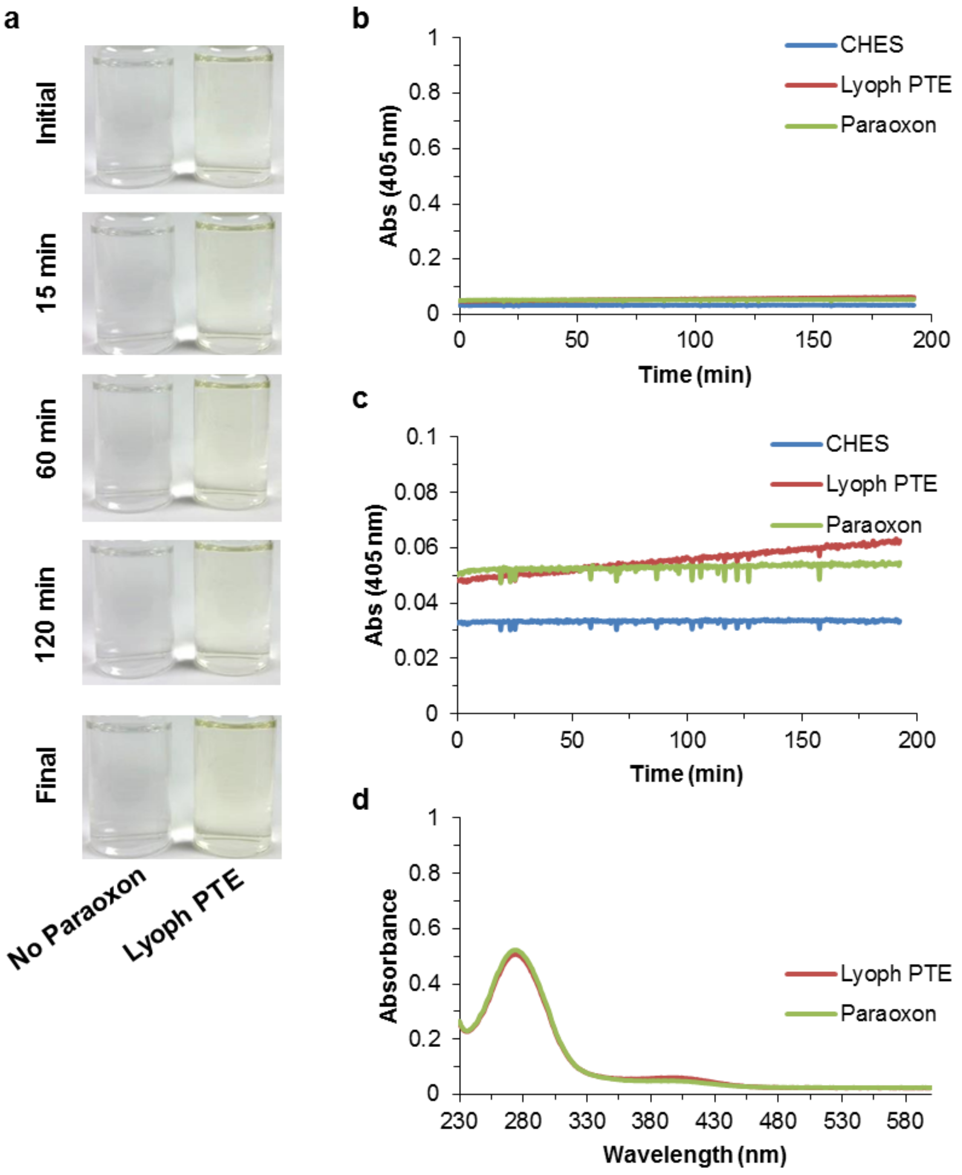


**Figure S9: Lyophilized free PTE long-term paraoxon remediation.** Larger-scale remediation of paraoxon utilizing lyophilized PTE. **a)** Lyophilized free PTE powder was added directly to paraoxon contaminated water in scintillation vials and pictures were taken over a 3 h period of time. **b)** Paraoxon degradation was monitored at 405 nm demonstrating very little remediation over the course of the experiment. **c)** Zoomed in representation of (b) to demonstrate that there was a very small amount of residual PTE activity (~1%) which is consistent with the retained PTE activity post lyophilization data presented in the text in Fig. 4. **d)** Absorbance spectrums were taken to demonstrate almost no conversion of paraoxon (280 nm) to *p*-nitrophenol (405 nm) at the end of the 3 h assay.

a

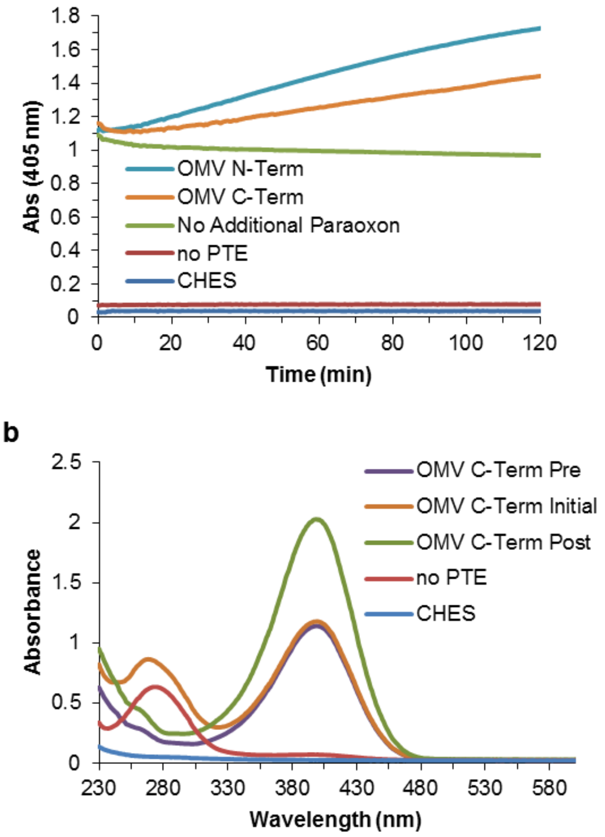


**Figure S10: Long-term paraoxon remediation.** After 4 days of RT storage a second remediation cycle utilizing the exact same samples from Fig. 5 were carried out with an equal quantity of paraoxon. **a)** Progress curves were monitored for paraoxon degradation at 405 nm with complete remediation utilizing the PTE/OMV$^{C}$ within 360 min. There was no breakdown of paraoxon in the absence of PTE or in the absence of an additional bolus of paraoxon. **b)** Absorbance spectrums were taken after 8 h to demonstrate complete conversion of paraoxon (280 nm) to p-nitrophenol (405 nm). [OMV C-Term Pre: spectrum prior to the addition of the second bolus of paraoxon, OMV C-Term Initial: spectrum directly following the addition of the second bolus of paraoxon, OMV C-Term Post: spectrum taken at the end of the experiment]**.** This experiment was conducted as such to demonstrate that packaged PTE-SC retains stability over many days at RT, in the presence of the paraoxon breakdown products, and despite varying initial concentrations of enzyme provides for complete conversion of the contaminant.
